# Integrating augmented reality technology in education: vector personal computer augmented reality

**DOI:** 10.12688/f1000research.72948.1

**Published:** 2021-09-30

**Authors:** Sin Yin Tan, Noel Nuo Wi Tay

**Affiliations:** 1 Faculty of Information Science and Technology, Multimedia University, Melaka, 75450, Malaysia

**Keywords:** Augmented Reality, Teaching and Learning, Learning Vectors, Online Class

## Abstract

**Background:** Educators often face difficulties in explaining abstract concepts such as vectors. During the ongoing coronavirus disease 2019 (COVID-19) pandemic, fully online classes have also caused additional challenges to using conventional teaching methods. To explain a vector concept of more than 2 dimensions, visualization becomes a problem. Although Microsoft PowerPoint can integrate animation, the illustration is still in 2-dimensions. Augmented reality (AR) technology is recommended to aid educators and students in teaching-learning vectors, namely via a vector personal computer augmented reality system (VPCAR), to fulfil the demand for tools to support the learning and teaching of vectors.

**Methods:** A PC learning module for vectors was developed in a 3-dimensional coordinate system by using AR technology. Purposive sampling was applied to get feedback from educators and students in Malaysia through an online survey. The supportiveness of using VPCAR based on six items (attractiveness, easiness, visualization, conceptual understanding, inspiration and helpfulness) was recorded on 5-points Likert-type scales. Findings are presented descriptively and graphically.

**Results:** Surprisingly, both students and educators adapted to the new technology easily and provided significant positive feedback that showed a left-skewed and J-shaped distribution for each measurement item, respectively. The distributions were proven significantly different among the students and educators, where supportive level result of educators was higher than students. This study introduced a PC learning module other than mobile apps as students mostly use laptops to attend online class and educators also engage other IT tools in their teaching.

**Conclusions:** Based on these findings, VPCAR provides a good prospect in supporting educators and students during their online teaching-learning process. However, the findings may not be generalizable to all students and educators in Malaysia as purposive sampling was applied. Further studies may focus on government-funded schools using the newly developed VPCAR system, which is the novelty of this study.

## Introduction

In the 21st century, the education system is slowly moving towards digital learning or online learning by integrating web 2.0 ^1^ and other technology tools in the teaching-learning process. Blended learning activities are integrated in the courses especially in higher education in Malaysia. These e-learning systems would encourage learners to learn at anytime and anywhere. Several courses related to blended learning such as flipped classroom, ^2^ massive open online courses (MOOCs) ^3^ are being conducted to upskill educators and support them by providing the facilities and devices. However, secondary and primary schools in Malaysia are still focusing on physical classes. Lately, the lockdown of the country due to the coronavirus disease 2019 (COVID-19) pandemic has caused all school, colleges and universities to conduct fully online classes in order to ensure that teaching-learning can take place without physical classes.

The emergence of technology is gradually changing the pedagogy of teaching-learning especially for mathematics subjects. Before such technology, educators wrote the equations on a black board to show the steps of the solution. This was followed by the use of portable overhead projectors. Up to now, educators have often used liquid crystal display (LCD) projectors to present Microsoft PowerPoint slides of the subject content. With the multimedia tools integrated with PowerPoint, it is much easier to stimulate students’ understanding on mathematics subject than using black/white board. ^4^ However, educators always feel challenged to illustrate the presentation of vectors and planes to students by using the traditional way such as white board or PowerPoint slide, especially when involving more than 2 dimensions. ^5^

In this study, augmented reality (AR) technology is introduced to support educators in their teaching-learning process especially when classes are conducted fully online. Feedback was being obtained from educators and students regarding the AR interface, understanding through visualization and inspiration in learning.

### Literature review

Augmented reality is one of the emerging technologies which bring great impact to different application domains. ^6- 9^ Different types of tools have been created by merging virtual objects with the real world in a scenario-based application. The interaction of the technology creates fun and gains users’ attention, such as AR mobile game Pokemon Go ^10^ which was a successful proof of concept of AR in year 2016 and attracted many users’ attention in the world especially the young people (predominantly 21-30 years old). ^11^

It is crucial to let students understand fundamental concepts before applying the knowledge in problem solving. Any misconceptions could lead to incorrect decisions. Hence, educators try to use effective teaching-learning methods to make sure students really understand. Sometimes, it is a great challenge to explain abstract contents to students especially for science, technology, engineering and mathematics (STEM) subjects. Therefore, educators have tried using different AR tools over the past two decades. ^7- 9, 12^ For example, in physics, the AR technology has been implemented in STEM education and showed positive effect in the process of teaching-learning abstract concepts such as forces, mass and other properties. ^13^ Besides that, an education tool was developed by Matsutomo *et al.* to observe the movement of magnetic fields using real-time visualization system ^14^ and Chen did a comparison between representation of AR and physical models in learning amino acids. ^15^ Nevertheless, Physical Education teachers have also revealed that AR is a great tool in improving human motion skill and preserving human health competence. ^16^ The improvement of AR technology and pedagogical methodology may allow learners to absorb the knowledge faster.

Regarding mathematics related research, several studies have looked at geometry especially for 3-dimension models, ^17^ but limited research has focused on vectors. The visualization of 3D geometry is somehow challenging for students. ^18, 19^ Construct3D was developed based on “Studierstube” in geometry education ^20^ and has been further applied in a PhysicsPlayground simulation. ^21^ Like geometry, vectors are a common abstract property in mathematics, which consists of direction and magnitude. Vectors are typically represented in a directed line with an arrow. Its quantities include displacement, velocity, position, force, and torque which are used in STEM subjects. Anablel *et al.* used gestures and body movement with the Kinect device in learning Euclidean Vectors. ^22^ To increase the mobility of users, Vectors AR3-APP was developed to support the educators and students in teaching and learning three-dimensional abstract concepts such as vectors and linear algebra whereas the learning module was developed in mobile apps. ^23^

AR systems can be developed in mobile apps, desktop computers, tabletops and other devices. One study revealed that users prefer more the use of a desktop AR system than mobile system in that the desktop AR system gained significant positive feedback in terms of responsiveness of the AR application. ^24^ In order to have better quality illustration by using smartphones, the users may need a higher specification smartphone which is costly. Hence, a new Vector PC AR system (VPCAR) will be proposed in this study to aid educators and students in their teaching-learning process with the following objectives.

**Objectives of this study:**

I. To attract the students' attention in learning vectors.
II. To make it easier for the students in understanding vectors.
III. To improve the students' visualization of the vectors’ representation.
IV. To strengthen the students’ conceptual understanding of vectors through visualization.
V. To inspire the students in learning vectors through its engagement and interactivity.
VI. To help the students and educators in learning/teaching vectors during the COVID-19 pandemic.

## Methods

### AR learning module development

**Figure 1.**
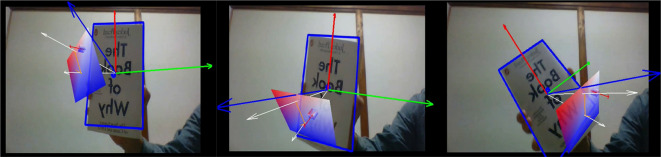
Illustration of vectors and plane by using Vector Personal Computer Augmented Reality.

Initially, the vectors’ learning module had been developed on a PC, where the vectors and plane were illustrated in a 3-dimensional coordinate system by using AR technology as illustrated in Figure 1. Python 3.6 and a laptop-integrated webcam are the main tools to develop the learning module with OpenGL3.1.5 and OpenCV4.1.0.25 library.

The AR vectors and planes were built so that they manifest themselves on patterned surface, like on top of a book whereas the vectors can be initially set by the users in the learning module. By moving the webcam, the users may view vectors from different perspectives. This learning module (a short video demonstration) was attached in the questionnaire. ^44^

### Questionnaire development

**Figure 2.**
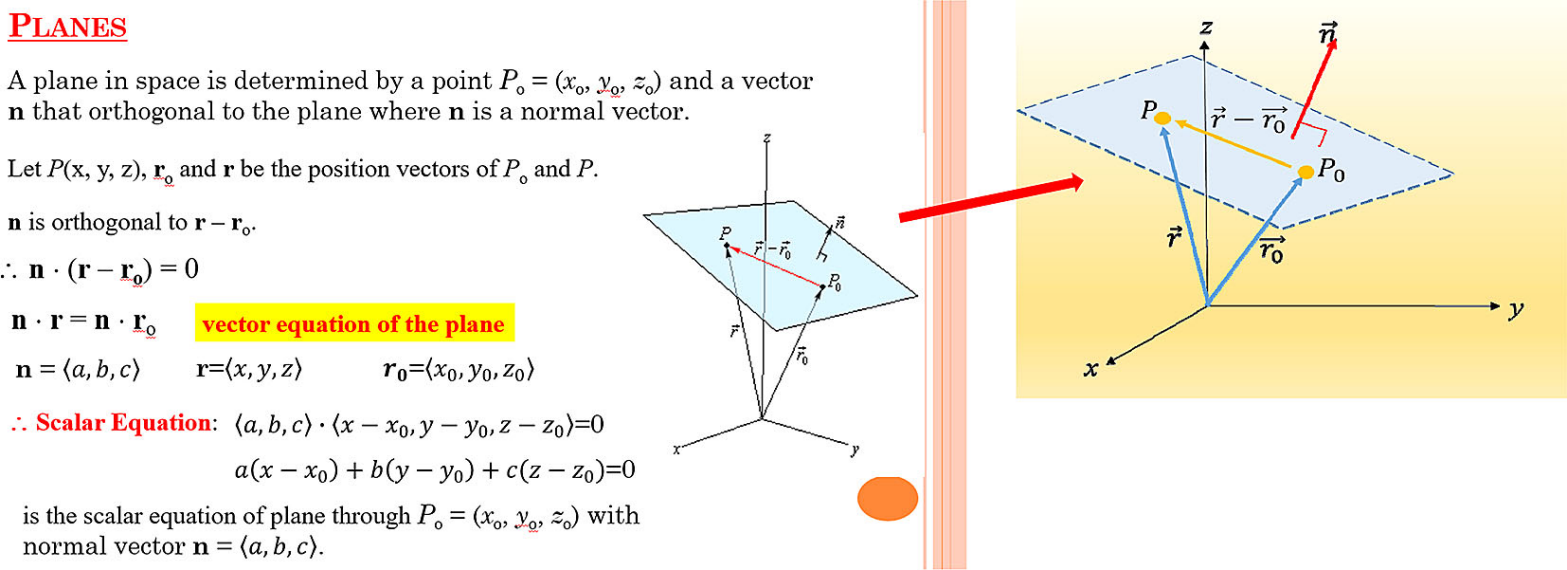
Illustration of vectors and plane by using Microsoft PowerPoint.

A questionnaire was designed based on the objectives of the study by using Google form to measure the supportiveness of incorporating AR technology in teaching and learning process during this pandemic. ^43^ It consisted of demographic information and the six measurement items based on the study objectives; attractiveness, easiness, visualization, conceptual understanding, inspiration and helpfulness. All items were measured by five-points Likert-type scale. The questionnaire was validated by an expert who has been teaching mathematic for more than 10 years and content validity was checked by using Pearson’s product moment test. ^25^ Cronbach’s alpha was used to measure the reliability of the questionnaire (discussed in the Results). A short video demonstration of vectors representation by using VPCAR and PowerPoint (duration: 2.12 minutes) was attached to the questionnaire. ^44^ Figures 1 and 2 were the screenshots. Ethics approval (EA1802021) was obtained from Multimedia University Malaysia, Technology Transfer Office (TTO). In addition, we obtained written informed consent from the respondents when completing the questionnaire.

### Data collection

Primary data from 167 students and 71 educators in Malaysia was collected through email and social media invitation who are in our contacts such as Facebook, WhatsApp over four weeks from March to April 2021. A purposive sampling method was applied in the selection of target population of students and educators from secondary schools to universities in Malaysia because directly approaching the educators and students may provide more valid feedback. ^26^ However, the result may not be able to generalise due to purposive sampling was applied that causes possibility of bias concern and the sample size was referred to recent studies in the same domain. ^27, 28^ The questionnaire was sent via email. Descriptive results were presented graphically and in tabular format. Since the feedback was collected in ordinal form, therefore Mann-Whitney U Test and Chi-square test were applied. The analysis was done by using SPSS 26. Microsoft Excel may be used as open-access alternative.

## Results

### Demographic profile of students and educators

Table 1 shows the demographic profile of the 167 students and 71 educators who took part. ^42^ The majority of students were aged 18 to 25 years old (95%) and studying at diploma level (74.25%); the majority of educators were aged 31 to 40 years old (57.75%) and teaching higher education level (94.37%). Participants were mainly from private institutes (85.63%, students; 92.96%, educators). In total, 86.83% of students had learned about vectors before and 54.93% of educators taught vectors with an average teaching experience of 14 years.

**Table 1.** Frequency and percentage distributions of demographic profile of students and educators.

Characteristics	Students		Educators	
	n = 167	%	n = 71	%
**Age (years)**				
Under 18	3	1.80	-	-
18 ~ 25	160	95.81	-	-
26 ~ 30	3	1.80	2	2.82
31 ~ 40	1	0.60	41	57.75
41 ~ 50	-	-	24	33.80
51 ~ 60	-	-	4	5.63
**Sex**				
Female	41	24.55	51	71.83
Male	126	75.45	20	28.17
**Studying/Teaching Institute**				
Government	24	14.37	5	7.04
Private	143	85.63	66	92.96
**Studying/Teaching Education Level**				
Secondary School	2	1.20	4	5.63
Foundation/STPM	1	0.60	67	94.37
Diploma	124	74.25		
Degree	39	23.35		
Postgraduate	1	0.60		
**Learn/Teach Vector**				
Yes	145	86.83	39	54.93
No	22	13.17	32	45.07
**Learning/Teaching experience**				
Secondary School	115	68.86	Mean=14.07 Max=30 Median=13 Min=5 Mode=10 SD=5.17	
Higher Education	30	17.96		
Not Applicable	22	13.17		

**Figure 3.**
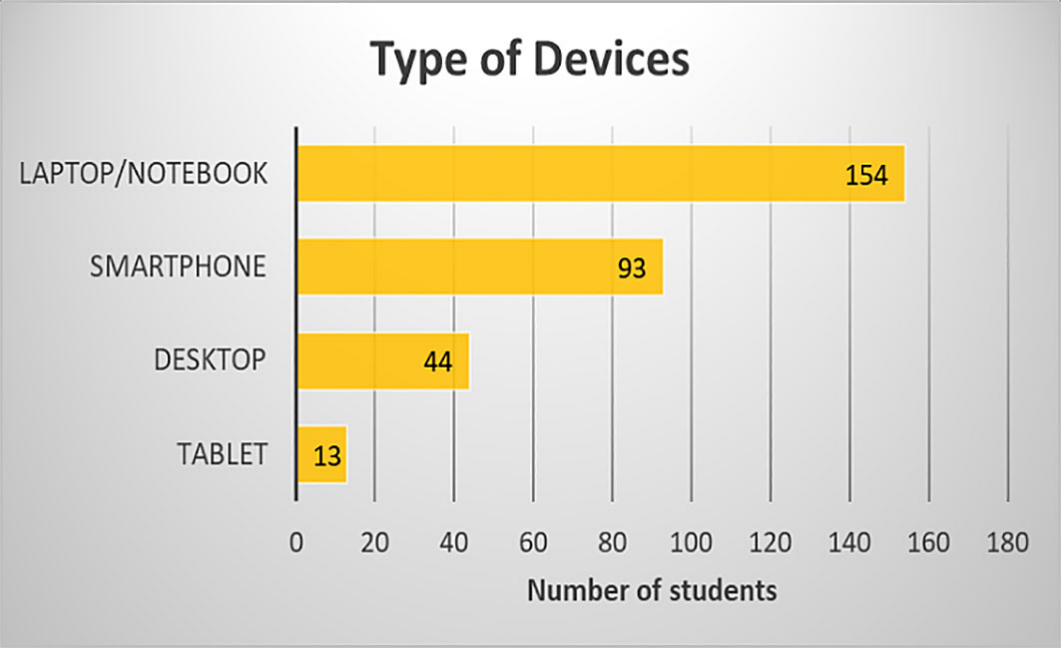
Type of devices used by students.

**Figure 4.**
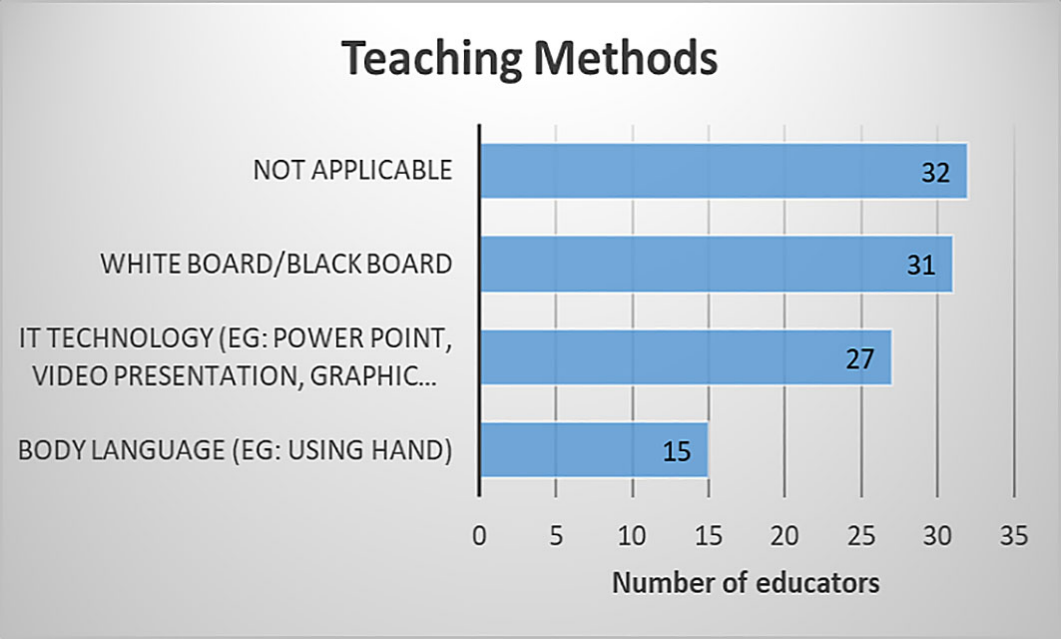
Teaching method used by educators.

During the COVID-19 pandemic, teaching-learning has been conducted fully online. Most students used a laptop or notebook (92.22%), followed by a smartphone (55.69%), represented in Figure 3. As shown in Figure 4, out of 39 educators, 79.49% of them used a white board or black board to teach vectors while 69.23% used IT technology such as PowerPoint, video presentation, and other graphic software. Less than 40% of them used body language to teach vectors. Especially for online classes, it is not easy to use body language to present vectors via webcam. Hence, VPCAR could be of great help to them.

**Figure 5.**
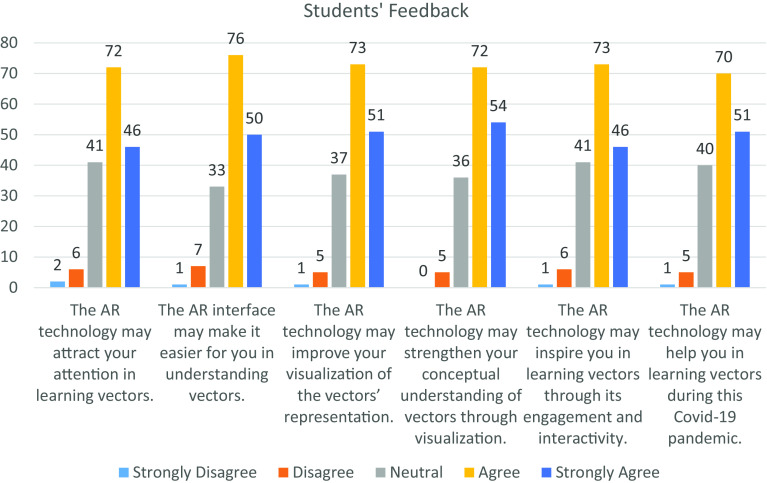
Feedback from students. AR = augmented reality; COVID-19 = coronavirus disease 2019.

**Figure 6.**
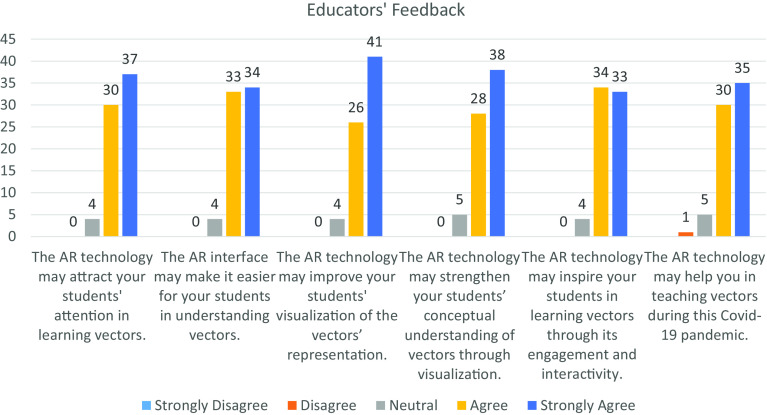
Feedback from educators. AR = augmented reality; COVID-19 = coronavirus disease 2019.

Figures 5 and 6 illustrate the feedback from the students and educators towards AR technology based on the six measurement items. Overall, the students and educators had provided a significant positive feedback towards incorporating AR technology in the teaching-learning process. From the diagram, the distribution of each measurement item among the students and educators are left skewed and J-shape respectively, which shows a significant difference in distribution at the 5% level of significance by using Mann-Whitney U Test. In Table 2, Cronbach’s alpha of the six measurement items for students and educators are more than 0.9 which indicates an excellent internal consistency in measuring the supportiveness on using AR technology in teaching-learning process. The content validity of the questionnaire was measured statistically by Pearson product moment correlation coefficient, *r*, which shows that all the measurement items is valid. In addition, the feedback was also measured by using mean score which is shown in Table 3. The mean score for each measurement item for educator category is higher than student category. This shows that the educators are more in support than the students in implementing AR technology tools in teaching-learning vectors.

**Table 2.** Cronbach’s alpha and Pearson product moment correlation coefficient, *r.* AR = augmented reality; COVID-19 = coronavirus disease 2019.

Measurement items	Students (Cronbach’s Alpha = 0.944)		Educators (Cronbach’s Alpha = 0.952)	
	*r*	*p*-value	*r*	*p*-value
The AR technology may attract your/your students' attention in learning vectors.	0.848	0.00	0.919	0.00
The AR interface may make it easier for you/your students in understanding vectors.	0.893	0.00	0.933	0.00
The AR technology may improve your/your students' visualization of the vectors’ representation.	0.921	0.00	0.913	0.00
The AR technology may strengthen your/your students’ conceptual understanding of vectors through visualization.	0.890	0.00	0.892	0.00
The AR technology may inspire you/your students in learning vectors through its engagement and interactivity.	0.889	0.00	0.877	0.00
The AR technology may help you in learning/teaching vectors during this COVID-19 pandemic.	0.863	0.00	0.869	0.00

**Table 3.** Feedback of the six measurement items, measured on a 1-5 Likert-type scale. SD = standard deviation; AR = augmented reality; COVID-19 = coronavirus disease 2019.

Categories	Students		Educators	
Measurement items	Mean	SD	Mean	SD
The AR technology may attract your/your students' attention in learning vectors.	3.92	0.88	4.46	0.61
The AR interface may make it easier for you/your students in understanding vectors.	4.00	0.85	4.42	0.60
The AR technology may improve your/your students' visualization of the vectors’ representation.	4.01	0.84	4.52	0.61
The AR technology may strengthen your/your students’ conceptual understanding of vectors through visualization.	4.05	0.81	4.46	0.63
The AR technology may inspire you/your students in learning vectors through its engagement and interactivity.	3.94	0.85	4.41	0.60
The AR technology may help you in learning/teaching vectors during this COVID-19 pandemic.	3.99	0.85	4.39	0.69

Typically, educators teach by using Microsoft PowerPoint nowadays. Table 4 shows that more than half of students (67.07%) and educators (76.06%) were keener to use AR technology during teaching-learning process. The findings revealed that most students are using a laptop or notebook to attend online classes, therefore PC learning module is more preferred than mobile apps learning module. Nearly 80% of educators keen to use PC AR tools. The positive association between learning/teaching method and learning module preferences was shown at 5% level of significance by using Chi-Square test with *p*-value = 0.021.

**Table 4.** Preferences of learning/teaching method and learning module.

Categories	Students		Educators	
Preferences	n = 167	%	n = 71	%
**Learning/** **Teaching Method**				
PowerPoint Slide	41	24.55	15	21.13
AR Technology	126	75.45	56	78.87
**Learning Module**				
Personal Computer	112	67.07	54	76.06
Mobile Apps	55	32.93	17	23.94

## Discussion

In summary, the finding showed significant positive feedback towards the incorporation of AR technology in teaching and learning vectors from educators and students based on the six measurement items. The visual aesthetic of a system is always the most important dimension in attracting the users’ attention. ^29^ The effort of creating a colourful and animated interface could let students feel more interested and focused during learning as previous research has found that some students consider this dimension encouraging in continuing using their website. ^30^ Thus, the colourful interface of AR could attract students’ attention of various ages such as those in higher education, secondary school, primary school etc. ^31, 32^

Furthermore, the main purpose of several augmented reality systems is targeting the improvement of the visualization of users ^14, 15, 20- 23^ as such it helps the users comprehend the fundamental concepts of particular content to avoid misunderstandings which was highlighted earlier in the literature review. AR tools are just an additional or supplementary method for explaining complex concepts which may be hard to be understood statically with our vision alone. Thus, the illustration of VPCAR make it easier for the students in understanding vectors through visualization as the students are able to view the vectors in different perspectives by moving their own PC’s webcam. The students’ visualization of vectors can therefore be improved compared to those who learn by using conventional teaching method. At the same time, AR may strengthen the students’ conceptual understanding of vectors. ^33^

Learning can be divided into learner-centred or instructor-centred learning. It is important to engage and get feedbacks from students by using a mixed approach so that the students won’t feel neglected. Educators always need to interact with the students so that the students won’t lost their interest in learning. Through this engagement, students will be inspired and motivated in learning. ^34^ AR technology can increase the engagement of students as reported in a previous study. ^35^ Thus, VPCAR may be able to inspire the students in learning vectors through its engagement and interactivity as shown by results of this study.

The feedback of students and educators are useful in determining their readiness in adopting AR technology. AR technology is easy to customise with low preparation overheads (costs) and it supports animation. Nowadays, PCs have become an essential electronic device in our lifestyle especially for working adults and students. VPCAR, which is developed for PC, requires less effort to use compared to other AR systems. As supported by the students and educators, VPACAR certainly provides great support to them in learning or teaching vectors during this COVID-19 pandemic.

### Limitations

In this study, the findings may not represent all students and educators in Malaysia because a purposive sampling method was applied. Due to limited network connection, most respondents were from private institutes. For further study, government secondary school students shall be included to distinguish the usefulness of VPCAR perception at different educational institutes.

## Conclusions

The outbreak of COVID-19 caused classes to be conducted fully online all over the world ^36^ and several works studied the challenges and issues faced by the educators and students during the pandemic. ^37- 39^ In this study, VPCAR was applied to an online class domain that encounters the problems faced by educators and students in learning or teaching vectors. The positivity of survey responses based on the six measurement items: attractiveness, easiness, visualization, conceptual understanding, inspiration and helpfulness, with the range of means from 3.92 to 4.52 out of 5, indicates that VPCAR is able to achieve the objective of this study from the aspect of educators and students. In addition, at least 75% of the educators and students preferred to use AR technology compared to PowerPoint slides which indicates that both educators and students are ready in adopting the AR technology in teaching-learning process.

With the merit of AR technology, several challenges and issues still need to be addressed. ^40, 41^ The system provides in real time visualization and requires internet connection. Hence, rural area students who lack of this facility may be unable to utilise this system efficiently. Increased internet access will give the opportunity to enhance this new technology. Furthermore, educators may also record the demonstration, so students are still able to learn it offline. The newly developed VPCAR system is the novelty in this study which is beneficial for the educators and students.

## Data availability

### Underlying data

Figshare: Integrating AR Technology in Teaching and Learning Vectors. http://doi.org/10.6084/m9.figshare.14864853. ^42^

### Extended data

Figshare: VPCAR Questionnaire 2021. http://doi.org/10.6084/m9.figshare.16608448. ^43^

Figshare: VPCAR Video Demostration 2021. http://doi.org/10.6084/m9.figshare.16649029. ^44^

Data are available under the terms of the Creative Commons Zero “No rights reserved” data waiver (CC0 1.0 Public domain dedication).

## Author endorsement

Prof. Ts. Dr. Lau Siong Hoe confirms that the author has an appropriate level of expertise to conduct this research, and confirms that the submission is of an acceptable scientific standard. Prof. Ts. Dr. Lau Siong Hoe declares they have no competing interests. Affiliation: Faculty of Information Science and Technology, Multimedia University, Jalan Ayer Keroh Lama, 75450 Bukit Beruang, Melaka, Malaysia.
