# Supplementary material for: Integrating augmented reality technology in education: vector personal computer augmented reality
Source: F1000Res. 2022 Mar 21;10:987. Originally published 2021 Sep 30. [Version 2] doi: 10.12688/f1000research.72948.2 (PMC10520515; doi:10.12688/f1000research.72948.2)
Supplement: Peer review history

# Reviewer report: Reviewer response for version 2

**DOI:** 10.5256/f1000research.122430.r201002

**Published:** 2023-09-25

**Authors:** Nur Idawati Md Enzai

**Affiliations:** 1 Universiti Teknologi MARA, Shah Alam, Selangor, Malaysia

This paper presents the implementation of AR to visualize vectors in education. Feedback from the users is also obtained and analyzed. I have a similar work implementing AR for computer hardware subjects but not as advanced as this work. This work utilizes Python and OpenGL. Therefore, I find the topic very interesting and look forward to future works to be executed. However, I hope the method of developing AR can be elaborated more, maybe it can be visualized in the form of flow chart, if possible.

Is the work clearly and accurately presented and does it cite the current literature?

Yes

If applicable, is the statistical analysis and its interpretation appropriate?

I cannot comment. A qualified statistician is required.

Are all the source data underlying the results available to ensure full reproducibility?

Yes

Is the study design appropriate and is the work technically sound?

Yes

Are the conclusions drawn adequately supported by the results?

Yes

Are sufficient details of methods and analysis provided to allow replication by others?

Yes

Reviewer Expertise:

Computer Networking, Engineering Education

I confirm that I have read this submission and believe that I have an appropriate level of expertise to confirm that it is of an acceptable scientific standard.

# Reviewer report: Reviewer response for version 2

**DOI:** 10.5256/f1000research.122430.r128158

**Published:** 2022-03-22

**Authors:** Kenneth Y. T. Lim

**Affiliations:** 1 Office of Education Research, National Institute of Education, Singapore, Singapore

Thank you for investing time and effort in understanding the intent behind the suggestions from the earlier round of review.

 I am satisfied that you have addressed them to the best of your ability.

Is the work clearly and accurately presented and does it cite the current literature?

Yes

If applicable, is the statistical analysis and its interpretation appropriate?

Yes

Are all the source data underlying the results available to ensure full reproducibility?

Yes

Is the study design appropriate and is the work technically sound?

Yes

Are the conclusions drawn adequately supported by the results?

Yes

Are sufficient details of methods and analysis provided to allow replication by others?

Yes

Reviewer Expertise:

AR in learning

I confirm that I have read this submission and believe that I have an appropriate level of expertise to confirm that it is of an acceptable scientific standard.

# Response

**Published:** 2022-03-23

**Authors:** Sin Yin Tan

**Affiliations:** Multimedia University, Malaysia

Thank you for taking the time and effort to review our manuscript.

# Reviewer report: Reviewer response for version 1

**DOI:** 10.5256/f1000research.76561.r99917

**Published:** 2021-12-09

**Authors:** Deepti Prit Kaur

**Affiliations:** 1 Chitkara University Institute of Engineering and Technology, Chitkara University, Chandigarh, Punjab, India

In general, the research presented here is interesting. The authors have developed a mechanism for visualizing and understanding the concept of vectors in a 3D coordinate system through Augmented Reality. However, the paper lacks clarity in many places, which can be fixed with major revisions.

1. The authors are encouraged to explain why augmented reality technology is needed for understanding these concepts when some online interactive simulators are already available for the same purpose.
2. There are many applications existing for the use of AR in geometry. Authors could include more literature with respect to existing applications. Also, a research gap may be highlighted with respect to the reviewed literature.
3. The contribution in technical development is not clear. The only specifications given are “Python 3.6 and a laptop-integrated webcam are the main tools to develop the learning module with OpenGL3.1.5 and OpenCV4.1.0.25 library.” In the methods section. It is not mentioned whether the system uses marker-based or marker-less AR. What can (or can’t) be done with this application? Were any usability principles considered for application design?
4. It is not clear whether there is any way to perform the calculations on vectors using the developed application. How are the inputs given/how are the coordinates shown? These must be specified.
5. Some language corrections are needed. Also, at one place (in discussion section), VPACAR is written in place of VPCAR.
6. This research mainly focuses on the feedback of educators and students (as evident from Figures 5-6, and Tables 2-4) on the use of application with respect to six measurement items and user's preferences of learning/teaching methods. The efficacy of application in student’s conceptual understanding must be found to meet objective IV of the study.

Is the work clearly and accurately presented and does it cite the current literature?

Yes

If applicable, is the statistical analysis and its interpretation appropriate?

I cannot comment. A qualified statistician is required.

Are all the source data underlying the results available to ensure full reproducibility?

Yes

Is the study design appropriate and is the work technically sound?

Partly

Are the conclusions drawn adequately supported by the results?

Partly

Are sufficient details of methods and analysis provided to allow replication by others?

Partly

Reviewer Expertise:

Augmented Reality, Engineering Education Research

I confirm that I have read this submission and believe that I have an appropriate level of expertise to confirm that it is of an acceptable scientific standard, however I have significant reservations, as outlined above.

# Response

**Published:** 2022-03-15

**Authors:** Sin Yin Tan

**Affiliations:** Multimedia University, Malaysia

Thank you so much for your valuable comments. 

 To address the comments that you had highlighted:

1. The AR concept is explained as such "AR can add an extra element of interaction, where user can treat it as if there is an actual object in front of them through visual-motor interaction". We added more explanation in the 'Introduction', the third paragraph in the paper.
2. Literature review had been extended in the third and fourth paragraph. The research gap is highlighted in the fifth paragraph, in the literature review.
3. We are using marker-less AR. Given a patterned flat surface, landmark features are extracted. Through homography, 3D perspective is inferred to generate the visualization. We added details in the 'Methods', in the 'AR learning module development'.
4. Currently, simple calculations can be performed. Position vectors and normal vectors on a plane can be presented in a 3-dimensional cartesian coordinate system by modifying the related vectors to be visualized in a file with scripts. Scripts is used instead of user interface as the user interface is under-developed. The output of this learning module was recorded as a short video demonstration.
5. We changed 'VPACAR' to 'VPCAR'.
6. To meet the objective IV of the study, several approaches can be used to measure the conceptual understanding of students such as qualitative and quantitative feedback. Educators commonly access the students’ understanding based on the correctness of answers. But it is challenging for educators to provide the quality feedback for each student during class especially for large group classroom. Hence, this study obtained the quality feedback of educators and students’ which provides measurement in Likert scale. It is crucial to obtain feedback from both parties to continue the suitable activities of teaching and learning in the class.

# Reviewer report: Reviewer response for version 1

**DOI:** 10.5256/f1000research.76561.r96142

**Published:** 2021-10-06

**Authors:** Kenneth Y. T. Lim

**Affiliations:** 1 Office of Education Research, National Institute of Education, Singapore, Singapore

I appreciate the opportunity to review your manuscript, and have the following suggestions for your consideration:

- The manuscript is clearly structured in an orthodox manner. To refine its intelligibility, I draw your attention to minor conjunctive phrases which are inappropriately used. For example, in the Literature Review, the second paragraph, the fifth sentence begins with 'Therefore'. The use of 'therefore' is not appropriate unless you first establish a connection between the use of AR tools and how they might address challenges in the explanation of abstract concepts. In the same paragraph, you use 'Nevertheless'. again, this word is inappropriate because all the cited works in this paragraph point towards positive outcomes, and therefore using 'nevertheless' makes little sense.
- In the third paragraph of the Literature Review, you say that the visualisation of 3D geometry is "somehow challenging". To your credit, you offer relevant cited works. Do please expand on them (at least briefly) because it is not appropriate in an academic paper to use 'somehow', when cited works can be elaborated upon to (at least partially) account for the 'somehow'. In the same paragraph, I do not understand your use of "to increase the mobility of users". I suspect it might be to do with the learning module in the mobile app, but this needs to be clearly explained. I also believe the word "whereas" in the last sentence of this paragraph is wrongly used, or - if it is correctly used - then the contrast (implicit in 'whereas') must be (very) clearly articulated.
- In 'Methods': 'AR learning module development', I am not clear about how the "vectors can be set by the users in the learning module", when "the learning module" is a "short video demonstration".
- In 'Methods': 'questionnaire development', 'mathematic' should be 'mathematics'. In the same paragraph, "Figures 1 and 2 were" should be rendered in the present tense.
- In 'Results': 'demographic profile'. In the second paragraph, you introduce the role of what you term 'body language' in the teaching and learning of 3D concepts such as vectors. While I understand implicitly what you refer to, I would like to suggest that 'body movement' / 'embodied action' be more appropriate than 'body language' (which has quite a different meaning). Second, still on this topic, while I understand what you mean, please try to find at least one or two literature-supported studies about embodied action / bodily movement in the teaching / learning of 3D concepts (such as vectors). This would strengthen your argument, because currently there is no prior context for you to abruptly introduce 'body language' at this stage of your manuscript.
- Still in this section, in the fourth paragraph, please insert 'are' in "nearly 80% of educators are keen".
- In the first paragraph of 'Discussion', you refer to "the colourful interface of AR", with the definite article "the". the use of color in the UI of AR is not necessarily a given, so please do not use the definite article ("the"). color is a possible attribute of UI in AR.

- In the third paragraph of 'Discussion', please correct the informal contraction "won't" to "will not". This occurs twice in the paragraph. Please also correct "won't lost" to "will not lose".

Is the work clearly and accurately presented and does it cite the current literature?

Yes

If applicable, is the statistical analysis and its interpretation appropriate?

Yes

Are all the source data underlying the results available to ensure full reproducibility?

Yes

Is the study design appropriate and is the work technically sound?

Yes

Are the conclusions drawn adequately supported by the results?

Yes

Are sufficient details of methods and analysis provided to allow replication by others?

Yes

Reviewer Expertise:

AR in learning

I confirm that I have read this submission and believe that I have an appropriate level of expertise to confirm that it is of an acceptable scientific standard, however I have significant reservations, as outlined above.

# Response

**Published:** 2022-03-15

**Authors:** Sin Yin Tan

**Affiliations:** Multimedia University, Malaysia

Thank you so much for your valuable comments. 

 To address the comments that you had highlighted:

- In the Literature Review, the second paragraph, the fifth sentence begins with 'Therefore' and 'Nevertheless', we will modify the English accordingly.
- In the third paragraph of the Literature Review, the challenges will be elaborated as such "it is hard for students to picture 3D spatial situation given only 2D figures." and related studies are cited. For the second point, the sentence will be improved as such:
   
  - To improve 3D visualization through visual-motor manipulation, Vectors AR3-APP was developed to support the educators and students in teaching and learning three-dimensional abstract concepts such as vectors and linear algebra where the learning module was developed in mobile apps.
- In 'Methods': 'AR learning module development', the AR learning module is a program where users will set the function of the vectors. The short video is merely a means to show the output for this paper. We have clarified it in the second paragraph.
- In 'Methods': 'questionnaire development', we changed 'mathematic' to 'mathematics' and "Figures 1 and 2 were" to "Figures 1 and 2 are"..
- In 'Results': 'demographic profile', we modified ‘body language’ to ‘body movement’ where body movements include gestures, posture, head and hand movements or whole body movements. Related studies are cited.
- In 'Results': in the fourth paragraph, we inserted 'are' in "nearly 80% of educators are keen".
- In the first paragraph of 'Discussion': we changed "the colourful interface of AR" to "colourful interface of AR".
- In the third paragraph of 'Discussion': we changed "won't" to "will not". and "won't lost" to "will not lose".
